# Influences of graphene oxide support on the electrochemical performances of graphene oxide-MnO_2 _nanocomposites

**DOI:** 10.1186/1556-276X-6-531

**Published:** 2011-09-27

**Authors:** Huanping Yang, Jian Jiang, Weiwei Zhou, Linfei Lai, Lifei Xi, Yeng Ming Lam, Zexiang Shen, Bahareh Khezri, Ting Yu

**Affiliations:** 1 Division of Physics and Applied Physics, School of Physical and Mathematical Sciences, Nanyang Technological University, 637371, Singapore, Singapore; 2 School of Materials Science and Engineering, Nanyang Technological University, Nanyang Avenue, Singapore 639798, Singapore; 3 Division of Chemistry and Biological Chemistry, School of Physical and Mathematical Sciences, Nanyang Technological University, 637371, Singapore, Singapore; 4 Department of physics, Faculty of Science, National University of Singapore, 117542 Singapore, Singapore

## Abstract

MnO_2 _supported on graphene oxide (GO) made from different graphite materials has been synthesized and further investigated as electrode materials for supercapacitors. The structure and morphology of MnO_2_-GO nanocomposites are characterized by X-ray diffraction, X-ray photoemission spectroscopy, scanning electron microscopy, transmission electron microscopy, Raman spectroscopy, and Nitrogen adsorption-desorption. As demonstrated, the GO fabricated from commercial expanded graphite (denoted as GO(1)) possesses more functional groups and larger interplane gap compared to the GO from commercial graphite powder (denoted as GO(2)). The surface area and functionalities of GO have significant effects on the morphology and electrochemical activity of MnO_2_, which lead to the fact that the loading amount of MnO_2 _on GO(1) is much higher than that on GO(2). Elemental analysis performed via inductively coupled plasma optical emission spectroscopy confirmed higher amounts of MnO_2 _loading on GO(1). As the electrode of supercapacitor, MnO_2_-GO(1) nanocomposites show larger capacitance (307.7 F g^-1^) and better electrochemical activity than MnO_2_-GO(2) possibly due to the high loading, good uniformity, and homogeneous distribution of MnO_2 _on GO(1) support.

## Introduction

As one of the green supercapacitor electrode materials, MnO_2 _shows potential to replace RuO_2 _due to its high specific capacitance, environmental compatibility, low cost, and abundance in nature. In general, the fabrication of MnO_2 _can be readily realized on large scale using traditional chemical co-precipitation methods [1,2]. However, MnO_2 _powders produced by these methods suffer some disadvantages, like low specific surface area, and thus low specific capacitance in most cases. To improve the electrochemical performance, the strategy of direct deposition of MnO_2 _on large-surface-area materials, such as carbon blacks, carbon nanotubes, activated or mesoporous carbons [3-9], is quite promising. Recently, graphene oxide (GO), a shining-star material, has been widely investigated as a suitable support for MnO_2 _loading [10,11]. Thanks to the large accessible surface area provided by GO, more ions can transport onto the material surface, achieving high electric-double-layer capacitance in aqueous electrolytes. Furthermore, nanostructured MnO_2 _modified on GO support can effectually prevent the aggregation of GO nanosheets caused by van der Waals interactions. As a result, the available electrochemical active surface area for energy storage can be greatly enhanced.

Structurally, a single-layer of graphite oxide, also called GO, consists of a honeycomb lattice of carbon atoms with oxygen-containing functional groups which are proposed to present in the form of carboxyl, hydroxyl, and epoxy groups [12,13]. These functional groups can enlarge the gap between adjacent GO sheets. For instance, the (002) diffraction peak of pristine graphite is located at approximately 26°, and the interplane distance is 0.34 nm. After oxidation of graphite, the diffraction peak shifts to a lower angle, indicative of a larger interplane gap. The functional groups and the larger interplane gap enable GO sheets to be easily decorated or intercalated by polymers, quantum dots, and metal/metal oxide nanoparticles (NPs), etc. [10,11,14-20], which are favorable and much desired for various applications. Nevertheless, until now, few reports have focused on the effects of functional groups and interplane gap of GO on the loading amount of quantum dots or metal/metal oxide nanoparticles, and so on.

In this work, we study the influences of GO supports on the electrochemical behavior of MnO_2_-GO nanocomposites. Two types of GO nanosheets, denoted as GO(1) and GO(2), made from commercial expanded graphite (CEG) and commercial graphite powder (CGP), respectively, are employed as MnO_2 _supports for comparative study. GO(1) nanosheets are proved to have more functional groups and larger interplane gap compared to GO(2) nanosheets, which might be capable of enhancing the loading amount of MnO_2_. As a result, MnO_2_-GO(1) nanocomposite exhibits higher energy and powder densities in neutral aqueous electrolytes.

## Experimental

### Materials

Commercial expanded graphite, commercial graphite powder, 98% H_2_SO_4_, 30% H_2_O_2_, potassium permanganate (KMnO_4_) and NaNO_3 _were used as received. Distilled water was used in all the processes of aqueous solution preparation and washing.

### Material characterization

Scanning electron microscopy images were obtained on a field-emission scanning electron microscope (FE-SEM JEOL JSM-6700F; JEOL, Tokyo, Japan). Transmission electron microscopy (TEM) analyses were carried out using an electron microscope (JEM 2010F; JEOL, Tokyo, Japan) operating at 120 kV. The Raman spectra were recorded using a WITEC-CRM200 Raman system (WITEC, Germany). The excitation source is 532-nm laser (2.33 eV). X-ray photoelectron spectroscopy (XPS) measurement, was carried out on a thermo scientific ESCALAB 250 (Thermo Fisher Scientific, UK). The nanocomposites X-ray diffraction (XRD) studies were charactered by a Bruker D8 ADVANCE XRD (Bruker AXS, Germany). Nitrogen adsorption-desorption experiments were investigated at 77 K on an automatic volumetric sorption analyzer (Quantachrome, NOVA1200; Micromeritics, USA). The surface area was calculated using the Brunauer-Emmett-Teller equation. Pore size distributions were calculated by the Barrett-Joyner-Halenda (BJH) method using the adsorption branches. Quantitative elemental determinations were performed by firstly dissolving the solid samples with a CEM Mars microwave digester (Matthews, NC, USA), followed by analysis with a Thermo Scientific iCAP 6000 series inductively coupled plasma optical emission spectroscopy (ICP-OES, Thermo Scientific, England).

### Synthesis of GO

Two kinds of graphite were used for synthesizing GO by a modified Hummers method [21-23]. In brief, 2 g of CEG (or CGP) and 1.5 g of NaNO_3 _were added into 150 mL of 98% H_2_SO_4 _solution in a flask which was immersed in an ice bath. Afterwards, 9 g of KMnO_4 _was slowly added in the solution. Meanwhile, the temperature of the mixed solution was maintained below 20°C for 2 h to avoid overheating and explosion. The mixture was stirred for 5 days. Then, 10 mL of 30% H_2_O_2 _was added into the solution in order to completely react with the remaining KMnO_4_, leading to a bright yellow solution. Finally, the resulting mixture was washed by 3% H_2_SO_4 _and H_2_O until the pH value of the solution was approximately 5-6. GO powder was obtained after freeze drying the suspension, labeled as GO(1)/GO(2).

### Synthesis of MnO_2_-GO nanocomposites

The MnO_2_-GO nanocomposites were prepared by an *in situ *reduction method [24]. The detailed procedure was as follows: 200 mg of GO(1) (or GO(2)) was blended with 150 mL of 0.02 M KMnO_4 _solution in a three-necked round-bottomed flask. The as-obtained mixture was refluxed at 120°C for 12 h with sustained magnetic stirring. The nanocomposites, labeled as MnO_2_-GO(1) (or MnO_2_-GO(2)), was then centrifuged, washed, and finally dried in air at 55°C overnight.

### Electrochemical measurement

The working electrode of the electrochemical capacitors was fabricated by mixing the nanocomposites (15 mg) with 15 wt.% acetylene black and 5 wt.% polytetrafluorene-ethylene binder of the total electrode mass. A small amount of ethanol was added to the mixture for more homogeneous paste. The mixture was then pressed onto nickel foam current collector (1.0 × 1.0 cm) (washed by acetone and 0.1 M HCl carefully before use) to make electrodes. Electrochemical characterizations were carried out in a conventional three-electrode cell with 1 M Na_2_SO_4 _as the electrolyte. A platinum foil and saturated Ag/AgCl electrode were used as the counter and reference electrode, respectively. All electrochemical measurements were conducted using CHI 660 electrochemical workstation.

## Results and discussion

**Figure 1:**
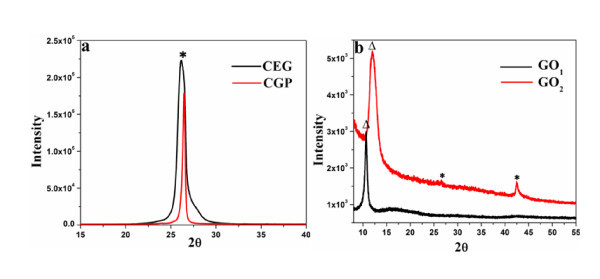
**XRD of (a) CEG and CGP; (b) GO(1) and GO(2)**. Diffraction peaks of graphite* and GO ^Δ^.

XRD patterns of CEG and CGP before and after oxidation are shown in Figure 1. As can be seen, the single peak at 2*θ *of 26.3° indicates the typical graphitic structure. Compared with CGP, the CEG shows a broad peak together with an upper shift of 0.4°, suggesting that CEG is amorphous and has a larger interlayer spacing. After chemical oxidation treatments, the GO(2) evolved from CGP still presents two XRD peaks corresponding to the typical graphitic faces, whereas the pattern of GO(1) from CEG only shows one peak. Thus, the XRD results reveal that the CEG can be more easily exfoliated than CGP. Two diffraction peaks of GO(2) at approximately 26.3° and approximately 42.5° can be clearly seen from Figure 1b, corresponding to (002) and (101) planes of graphitic framework, respectively [25,26]. All peaks are weak and broad, which illustrate an amorphous carbon framework. This occurs because the interlayer spacing of the few-layered graphene sheet is similar to that of normal graphite, indicating that CGP has been partially converted into GO. In addition, there is a main peak at 2*θ *of 12.1° in GO(2) and 10.6° in GO(1), corresponding to d-spacing of 0.73 and 0.83 nm, respectively. This peak is similar to the typical diffraction peak of GO and is a possible indication of the presence of inter-few-layered graphene containing defects [27]. GO(1) has a larger interplane gap than GO(2), revealing its higher oxidation degree compared to GO(2).

**Figure 2:**
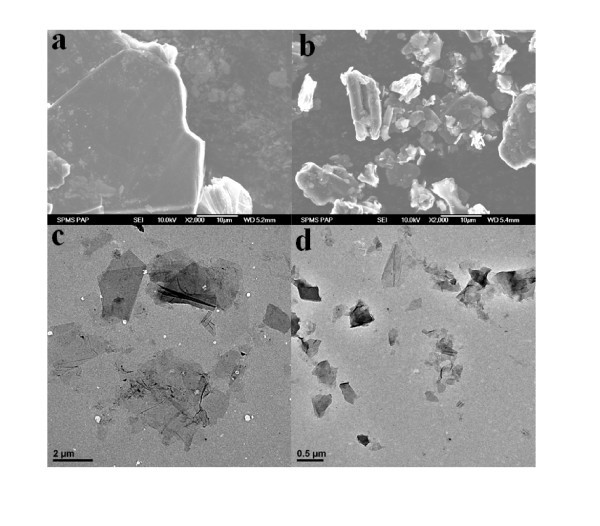
**SEM (a b) and TEM (c d) images of: (a) CEG, (b) CGP, (c) GO(1) and (d) GO(2)**.

**Figure 3:**
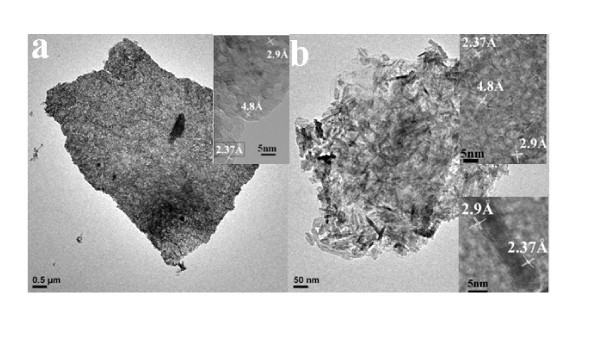
**TEM and HRTEM images of (a) MnO_2_-GO(1) and (b) MnO_2_-GO(2)**.

Figure 2a and 2b reveal the morphology differences between CEG and CGP. CEG has bigger graphite piece than CGP, which leads to larger GO(1) sheets than GO(2) (Figure 2c and 2d). Figure 3a and 3b show that the MnO_2_-GO(1) and MnO_2_-GO(2) still maintain the skeleton structure of GO with diameters around 10 μm, even larger than pristine GO (Figure 2c and 2d). This means that after hydrothermal reaction, the nanocomposites became agglomerate. The TEM observations show the prepared nano-MnO_2 _with morphology of nanorod and nanoflake uniformly decorated on the surface of the GO sheets. It is notable that both MnO_2 _nanorods and nanoflakes can be found on GO(2). While for GO(1) support, there are only MnO_2 _nanoflakes on its surface. Park and Keane have found that the strong epitaxial interaction between the catalytic species and the graphitic planes leads to a homogeneous distribution of the loaded Pd [28]. It is generally accepted that large interplanar spacing and high specific surface area of GO(1) would enhance the epitaxial interaction between nano-MnO_2 _and the GO planes. As a result, MnO_2 _nanoflakes can be distributed uniformly on GO(1) with smaller size than MnO_2 _nanorods and nanoflakes on the GO(2) [29].

The inset HRTEM images in Figure 3 show the lattice fringes of the MnO_2_-GO(1) and MnO_2_-GO(2) nanocomposites. Three distinct sets of lattice spacing of ca. 0.237, 0.29, and 0.48 nm are shown, corresponding to the (211), (001), and (200) planes of α-MnO_2_, respectively. The inset image in Figure 3a and 3 the upper inset image in Figure 3b present the orientation of the three epitaxial growths of MnO_2 _nanoflakes on GO(1) and GO(2). Both epitaxial growths for the formation of the MnO_2 _nanorods on GO(2) were revealed by the lower inset image in Figure 3b. The presence of clear lattice fringes in the HRTEM images confirms the crystalline nature of the α-MnO_2 _nanorodes and nanoflakes. The following Raman and XPS characterization also prove the polymorph of the MnO_2 _is α-MnO_2_.

**Figure 4:**
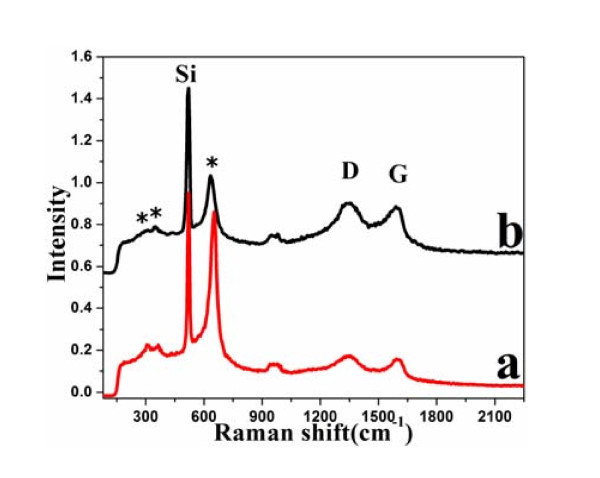
**Raman Spectra of (a) MnO_2_-GO(1) and (b) MnO_2_-GO(2)sheets**. (* α-MnO_2_)

The typical Raman spectra taken from different regions of the samples are shown in Figure 4. They present one diagnostic Raman scattering band of α-MnO_2_, approximately 643 cm^-1^, which belongs to A_g _spectroscopic species originating from breathing vibrations of MnO_6 _octahedra [30]. Two weak peaks recorded at approximately 305 and 360 cm^-1 ^corresponding to the bending modes of O-Mn-O were observed in the spectra of the nanocomposites, stemming from the formation of Mn_2_O_3 _or Mn_3_O_4 _induced by the laser heating [31]. The appearance of a strong A_g_-mode consists with our HRTEM result that the crystalline α-MnO_2 _has been readily formed on the GO support. Another two prominent peaks, D band (1,345 cm^-1^) and G band (1,597 cm^-1^), belong to GO [32-35]. From Figure 4, we can also see that the ratio of α-MnO_2 _to G is very different. MnO_2_-GO(1) has a larger α-MnO_2 _to G ratio than MnO_2_-GO(2), which means that the content of MnO_2 _in MnO_2_-GO(1) is higher than that in MnO_2_-GO(2). The Raman results are consistent with the inductively coupled plasma (ICP) and XPS results.

The element analysis was further studied by inductively coupled plasma (ICP) to prove the different amount of Mn in the nanocomposites. ICP-OES analysis of the concentrations of Mn in the nanocomposits confirmed that MnO_2_-GO(1) (230.3 mg g^-1^) has higher Mn content than MnO_2_-GO(2) (153.6 mg g^-1^), which will affect the morphology and electrochemical performances of the nanocomposites.

**Figure 5:**
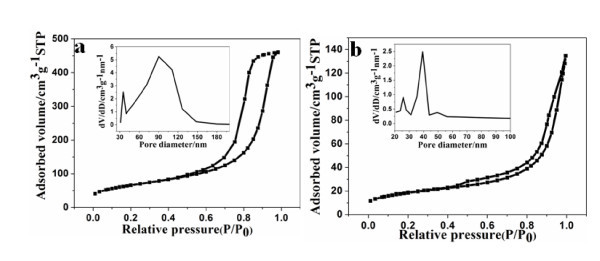
**Nitrogen adsorption-desorption isotherms of (a) MnO_2_-GO(1) and (b) MnO_2_-GO(2)**. The inset shows BJH pore-size distributions.

The nanocomposites obtained by using different GO sources have been further studied by the nitrogen adsorption-desorption measurements. As can be seen from Figure 5, all samples display a type-IV isotherm, indicating the mesoporous structure. Although MnO_2_-GO(1) and MnO_2_-GO(2) reveal the same type of the adsorption-desorption isotherm, their surface areas and pore size distributions are quite different. As for MnO_2_-GO(1), the specific surface area and the total pore volume are measured to be approximately 238.1 m^2 ^g^-1 ^and approximately 0.711 cm^3 ^g^-1^, which are correspondingly larger than those for MnO_2_-GO(2). Remarkably, these values are much higher than the data for MnO_2 _produced by traditional co-precipitation of KMnO_4 _and Mn^2+^in previous report [36]. The pore size distribution plots of MnO_2_-GO(1) and MnO_2_-GO(2) are calculated by BJH method, using desorption branch of N_2 _isotherms. MnO_2_-GO(1) and MnO_2_-GO(2) have comparable pore volumes. However, MnO_2_-GO(2) shows narrower pore size distribution than MnO_2_-GO(1), with a pore diameter range of 20-50 nm. The results clearly demonstrate that the graphite source has a significant effect on the microstructure of GO. The specific surface area and effective pores (8-50 Å) are reported to be effective to increase the double-layer capacitance of carbon and multiply the redox active sites for metal oxides loading. Therefore, the pseudo-capacitance will increase significantly. As a result, the unique structure could be useful to enhance the capacity of MnO_2_-GO(1) [24,37,38].

**Figure 6:**
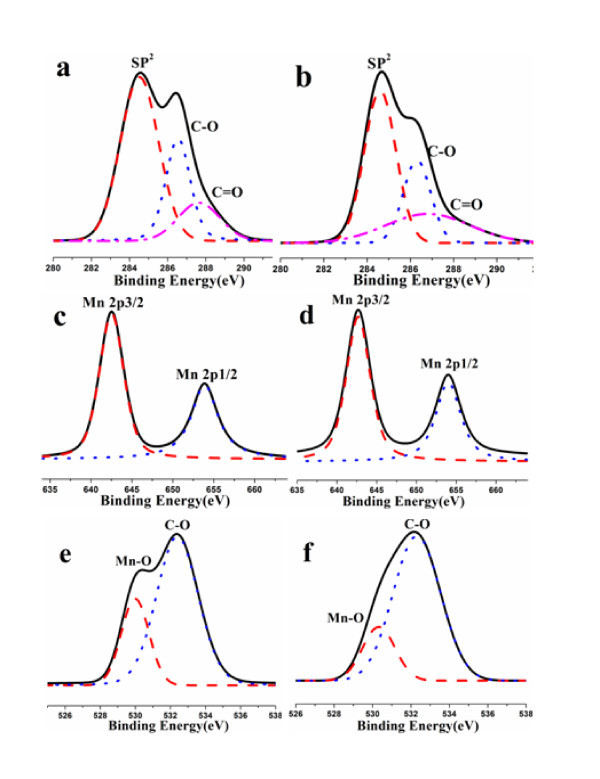
**C1s spectra: (a) GO(1), (b) GO(2)**. (c-d) Mn2p, (e-f) O1s spectra of MnO2-GO(1) and MnO2-GO(2).

The high-resolution XPS spectra further confirm the different oxygen contents in GO(1) and GO(2), manganese contents and carbon contents in MnO_2_-GO(1) and MnO_2_-GO(2). As shown in Figure 6a and 6b, the curve fitting yields three components at sp^2^-C (approximately 284.5 eV), C-O (hydroxyl and epoxy, approximately 286.5 eV), and C=O (carboxyl, approximately 288 eV), respectively [39-41]. The contribution of C=C band decreases from 50% for GO(2) to 40% for GO(1). An obvious broadening of C=C band is also observed, indicating a more disordered structure for GO(1), which agrees well with the XRD results.

The spectra in Figure 6c and 6d illustrate the existence of MnO_2 _by the peaks assigned to Mn 2p3/2 (642.7 eV) and Mn 2p1/2 (653.9 eV), respectively. They have a spin-energy separation of 11.2 eV, further confirming the presence of α-MnO_2 _in the nanocomposite [42,43].

Besides the oxygen (O 1*s*, 532.4 eV) signals from graphene sheets in Figure 6e and 6f, the O 1*s *peak observed at 530.0 eV is assigned to the oxygen bonded with manganese [44]. On the basis of the quantitative analysis of XPS data, the corresponding atomic ratios of Mn to C for MnO_2_-GO(1) and MnO_2_-GO(2) in the nanocomposite are estimated to be 1:1.61 and 1:1.81 by integrating the area of each element peak areas, with their relative sensitive factor taken into account as well. It is worth noting that most carbon atoms in graphene sheets have not been substituted by Mn. However, MnO_2_-GO(1) still has more replacement Mn position in the nanocomposite than MnO_2_-GO(2). All of the data further confirm the existence of α-MnO_2 _and the loading of MnO_2 _is higher in MnO_2_-GO(1) than that in MnO_2_-GO(2).

**Figure 7:**
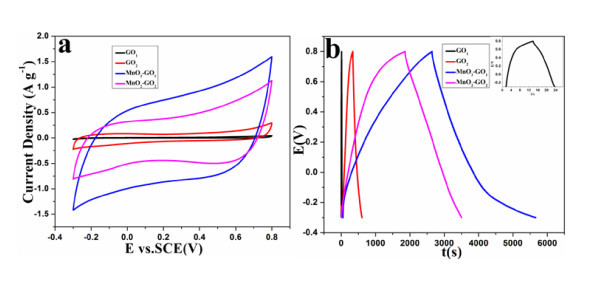
**(a) CVs and (b) galvanostatic charge/discharge curves of GO(1), GO(2), MnO_2_-GO(1), respectively**.

The electrochemical performances of the GO obtained from different graphite sources before and after loading MnO_2 _were investigated by cyclic voltammograms (CVs) and galvanostatic charge/discharge measurements in 1 M Na_2_SO_4 _solution between -0.3 and 0.8 V (Figure 7). From Figure 7a, it can be seen that the plots show an almost rectangular profile induced by an ideal capacitive behavior. GO shows lack of symmetry [43]. The poor electrochemical performance of GO is due to their poor electrical conductivity and low faradic reaction rate. However, the capacitance of GO(2) (21.39 F g^-1^) is higher than that of GO(1) (0.64 F g^-1^). Figure 7b shows the galvanostatic charge/discharge curves of the GO(1), GO(2), MnO_2_-GO(1), and MnO_2_-GO(2) at a current density of 100 mA g^-1^. After MnO_2 _loading, the capacity of MnO_2_-GO(1) is twice that of MnO_2_-GO(2) due to the high loading amount of MnO_2 _for GO(1) than that for GO(2).

**Figure 8:**
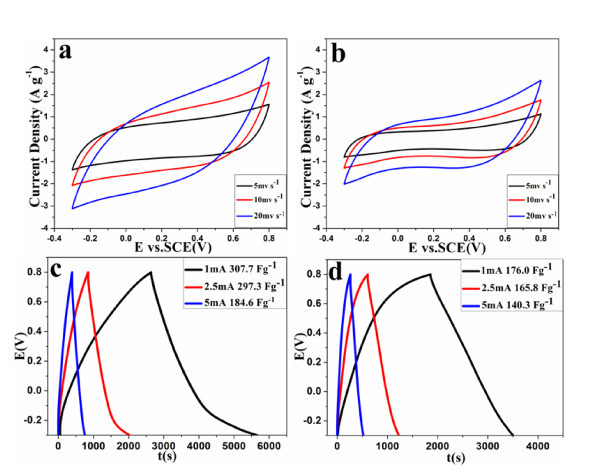
**(a-b) CVs and (c-d) galvanostatic charge/discharge curves of MnO_2_-GO(1) and (b) MnO_2_-GO(2), respectively**.

Figure 8a and 8b show the CV curves of MnO_2_-GO(1) and MnO_2_-GO(2) nanocomposites. MnO_2_-GO(1) shows the lack of symmetry at high scan rates (Figure 8b), which is probably due to pseudo-capacitance from MnO_2_[10,43]. Specific capacitances of the nanocomposites calculated at current densities of 100, 250, 500 mA g^-1 ^from the discharge curves are 176.0, 165.8, 140.3 F g^-1 ^for MnO_2_-GO(2) electrode, and 307.7, 297.3, 184.6 F g^-1 ^for MnO_2_-GO(1) electrode (Figure 8c and 8d). The MnO_2_-GO(1) electrode has almost twice the specific capacitances of MnO_2_-GO(2). The enhanced electrochemical performance of MnO_2_-GO(1) electrode is due to the high MnO_2 _loading by using the GO(1) with abundant surface functionalities. High loading and homogeneous distribution of MnO_2 _on graphene oxide surface are advantageous for graphene oxide network to transport ions in the pore system and increasing the MnO_2_-electrolyte interfacial area. Therefore, the excellent capability of GO(1) makes it attractive particularly for energy storage applications. Different GO precursors obviously have significant effect on the electrochemical capacitive performance before or after loading other nanomaterials. Thus, it is important to obtain highly porous and surface-functionalized graphene for supercapacitor applications.

## Conclusions

Based on the investigation of the chemical structure, morphology, and electrochemical behavior of MnO_2_-GO(1) and MnO_2_-GO(2), we conclude that the initial properties of GO have notable influences on the morphology and electrochemical activity of the GO-MnO_2 _nanocomposites. The GO synthesized from the CEG has more functional groups and lager interplane distance. Therefore, MnO_2 _nanoparticles can distribute homogeneously on GO(1) with high quantity. Because of the high surface area of MnO_2_-GO(1) and high loading efficiency of MnO_2_, the specific capacitance of MnO_2_-GO(1) is almost twice of MnO_2_-GO(2). The surface chemistry and structural properties of GO is of significant importance as nanoparticles carrier for various applications, such as catalyst, energy storage devices, etc.

## Competing interests

The authors declare that they have no competing interests.

## Authors' contributions

HPY carried out the total experiment and write the manuscript. JJ participated in the detection of the supercapacitor. WZ participated in the detection of the SEM. LL participated in the detection of the BET and XPS. LX carried out the TEM detection. YML participated in the statistical analysis. ZS participated in the statistical analysis. BK carried out the detection of ICP. TY participated in the design of the study and performed the statistical analysis. All authors read and approved the final manuscript.
